# Interaction between susceptibility loci in cGAS-STING pathway, MHC gene and HPV infection on the risk of cervical precancerous lesions in Chinese population

**DOI:** 10.18632/oncotarget.12399

**Published:** 2016-10-01

**Authors:** Di Xiao, Weihuang Huang, Meiling Ou, Congcong Guo, Xingguang Ye, Yang Liu, Man Wang, Baohuan Zhang, Na Zhang, Shiqi Huang, Jiankun Zang, Zixing Zhou, Zihao Wen, Chengli Zeng, Chenfei Wu, Chuican Huang, Xiangcai Wei, Guang Yang, Chunxia Jing

**Affiliations:** ^1^ Department of Epidemiology, School of Medicine, Jinan University, Guangzhou, Guangdong, China; ^2^ Department of Parasitology, School of Medicine, Jinan University, Guangzhou, Guangdong, China; ^3^ Key Laboratory of environmental exposure and health in Guangzhou, Jinan University, Guangzhou, Guangdong, China; ^4^ Family Planning Research Institute of Guangdong, Guangzhou, Guangdong Province, China; ^5^ Department of Pathophysiology, School of Medicine, Jinan University, Guangzhou, Guangdong, China

**Keywords:** cGAS-STING, MHC, SNP, interaction, cervical precancerous lesions

## Abstract

Human papillomavirus (HPV) infection is a definite risk factor for cervical cancer. Nevertheless, only some infected individuals actually develop cervical cancer. The cGAS-STING pathway in innate immunity plays an important role in protecting against HPV infection. Chen et al. described that the rs2516448 SNP in the MHC locus may affect susceptibility to cervical cancer, a finding that we attempted to replicate in a Chinese population. To investigate the effects of cGAS, STING and MHC polymorphisms on susceptibility to cervical precancerous lesions, 9 SNPs were analyzed in 164 cervical precancerous lesion cases and 428 controls. Gene-gene and gene-environment interactions were also evaluated. We found a significantly decreased risk of cervical precancerous lesions for the GG genotype of rs311678 in the cGAS gene (OR_adjusted_ = 0.40, 95% CI: 0.16−0.98). Moreover, MDR analysis identified a significant three-locus interaction model, involving HPV infection, age at menarche and rs311678 in cGAS. Additionally, a significant antagonistic interaction between HPV infection and rs311678 was found on an additive scale. In conclusion, our results indicate that the rs311678 polymorphism in the cGAS gene confers genetic susceptibility to cervical precancerous lesions. Moreover, the three-way gene-environment interactions further demonstrate that the rs311678 polymorphism in cGAS can significantly decrease the risk of HPV infection and the elder at menarche.

## INTRODUCTION

Cervical cancer is the third most common cancer among women, with approximately 530,000 new cases annually worldwide [1]. Moreover, with more than half a million new cases and 275,000 deaths per year, cervical cancer continues to constitute a major public health problem and particularly affects young women in developing countries [2, 3]. Cervical cancer develops through a multistep process, with three cervical intraepithelial neoplasia grades, 1 to 3 (CIN1-3) [4]. However, it will take several years, even decades, from pre-cancer to invasive cervical cancer, which offers us many opportunities for intervention. Therefore, early detection of cervical precancerous lesions and their causes likely contribute to reducing the incidence and mortality of cervical cancer.

Human papilloma virus (HPV) appears to be a necessary factor in the development of almost all cases (> 90%) of cervical cancer [5]. Molecular and epidemiologic studies have shown that persistent infection with high-risk HPV plays a role in the development of both cervical cancer and cervical precancerous lesions [6–8]. However, only some of the infected individuals develop cervical cancer. Although HPV is an essential factor for the transformation of cervical epithelial cells, it is not sufficient, and a variety of environmental and host factors influence the development of cervical cancer.

As a first line of antiviral defense, innate immune cells express cytokines such as type I interferons (IFN-I) that can activate and recruit innate and adaptive immune cells [9]. cGAMP synthase (cGAS) detects intracellular DNA and signals through the adapter protein stimulator of interferon genes (STING) to induce IFN-I, initiating the antiviral response to DNA viruses [10–12]. In principle, the microorganisms that can carry DNA into the host cytoplasm, such as DNA viruses (e.g., HSV-1, KSHV and adenovirus) [13–16], bacteria(e.g., Group B Streptococcus) [17], and retroviruses(e.g., HIV) [18] could potentially trigger the cGAS-STING pathway [12]. While it is still unknown how the activity of cGAS is regulated during host defense [19].

A recent study reported that one way that HPV may cause cancer is by targeting tumor suppressor proteins in the host [10, 20]. Oncogenes from HPV bind to the protein STING, allowing the virus to subvert the host's antiviral immunity and initiate infection, which, for an unlucky few, eventually causes cancer [20]. However, IFN-β serves as a signal linking innate and adaptive immunity that can defend against high-risk HPV activity [21, 22]. Analysis of SNP data from the 1000 Genomes Project revealed that there are four SNP-derived isoforms in hSTING, with R71H-G230A-R293Q (HAQ) in 20.4%, R232H in 13.7%, G230A-R293Q (AQ) in 5.2%, and R293Q in 1.5% of human population [23]. Furthermore, hSTING variation can affect innate immune signaling and HAQ STING is defective in stimulation of INFβ production [23, 24].

Thus, we hypothesized that individual genetic differences in the cGAS-STING pathway could influence the expression of IFN-I, which might influence the host antiviral response, and thus affect the susceptibility to developing cervical cancer [25, 26]. Furthermore, recent studies on the T allele of rs2516448 in the major histocompatibility complex (MHC) region, which increased the susceptibility to cervical cancer in a Swedish population, were published [27, 28]. The risk allele of rs2516448 in the first locus is in perfect LD with A5.1, a frameshift mutation of the MHC class I polypeptide-related sequence A gene(MICA). Chen et al. [28] reported that the MICA-A5.1 variant results in less membrane-bound MICA, which may affect immune activation and immune surveillance against HPV-infected cells, and increase the risk of cervical cancer development. Our study aimed to confirm this finding in Chinese women.

Investigating genetic differences and their interactions with the host's innate immune system could lead us to understand the precancerous lesions more comprehensively. In this study, nine polymorphisms in three candidate genes, including the cGAS gene (rs610913, rs311678, rs4032697, rs311675, rs9352000, rs7761170), the STING gene (rs1131769, rs7380824) and the MHC (rs2516448), were genotyped, and the possible associations of these SNPs with cervical precancerous lesions were investigated. Furthermore, we sought to explore the potential interactions between these SNPs and environmental factors in the etiology of cervical precancerous lesions in the Chinese population.

## RESULTS

### Population characteristics

The general characteristics are detailed in Table 1. Significant differences were found in age and HPV infection between the SIL cases and controls. No significant differences in the body mass index (BMI), number of term births, age at menarche, family history of cancer or genital cleaning after each intercourse were observed between the two groups.

**Table 1:** The distribution of demographic characteristics

Characteristics	Cases No = 164	Controls No = 428	*P*^a^
Age (years)	41.49 ± 7.97	42.93 ± 7.85	**0.046**
BMI (kg/m^2^)^a^	22.14 ± 2.82	22.36 ± 3.09	0.420
HPV infection			**< 0.001**
No	32 (19.5%)	251 (58.6%)	
Yes	132 (80.5%)	177 (41.4%)	
Number of term births	2.09 ± 1.07	2.04 ± 0.97	0.555
Age at menarche (years)	15.12 ± 1.97	14.93 ± 1.67	0.257
Family history of cancer			0.372
No	164 (100.0%)	421 (98.8%)	
Yes	0 (0%)	5 (1.2%)	
Genital cleaning after each intercourse			0.064
never	57 (36.5%)	178 (42.6%)	
occasional	47 (30.1%)	141 (33.7%)	
frequently	52 (33.3%)	99 (23.7%)	
The initial pregnancy of age (years)	23.94 ± 3.52	24.11 ± 3.31	0.572

Data are shown using means ± standard deviation for continuous factors.

a The χ^2^ test for categorical variables and student *t*-test for continuous variables

b BMI (kg/m^2^), body mass index. Bold values are statistically significant.

### Association between cGAS, STING and MHC polymorphisms and cervical precancerous lesions

As shown in Table 2, the association between rs311678 in cGAS and cervical precancerous lesions was significant under the allelic model (G vs A, *P* = 0.048), while the other eight SNPs were not found to be associated with the risk of cervical precancerous lesions.

**Table 2:** The association of the allele in cGAS, STING and MHC genes with the risk of cervical precancerous lesions

Gene	SNPs	Allele	Cases	Controls	OR (95% CI)	*P*
			(*n* = 164)	(*n* = 428)		
cGAS	rs610913	C	82	190	1 (Ref)	
		A	166	442	0.87 (0.64–1.19)	0.386
	rs311678	A	190	450	1 (Ref)	
		G	72	234	0.73 (0.53–1.00)	**0.048**
	rs4032697	A	306	778	1 (Ref)	
		G	8	29	0.70 (0.32–1.55)	0.379
	rs311675	T	300	760	1 (Ref)	
		A	14	43	0.83 (0.45–1.53)	0.541
	rs9352000	T	294	762	1 (Ref)	
		G	16	45	0.92 (0.51–1.66)	0.785
	rs7761170	G	218	556	1 (Ref)	
		T	65	164	1.01 (0.73–1.40)	0.948
STING	rs1131769	G	264	694	1 (Ref)	
		A	34	80	1.12 (0.73–1.71)	0.610
	rs7380824	C	98	284	1 (Ref)	
		T	142	364	1.13 (0.84–1.53)	0.424
MHC	rs2516448	G	208	532	1 (Ref)	
		A	67	168	1.02 (0.74–1.41)	0.905

Bold values are statistically significant.

The association of 9 SNPs in the cGAS-STING signaling pathway and MHC gene with cervical precancerous lesion risk is shown in Supplementary Table S4. We found that individuals with the GG genotype in cGAS rs311678 had a 60% decreased risk of developing cervical precancerous lesions (OR_adjusted_ = 0.40, 95% CI = 0.16–0.98, *P* = 0.045) compared with those with the AA wild-type, while the other 8 SNPs were not observed to be relevant to the risk of cervical precancerous lesions.

### Haplotype analysis

The associations between cGAS and STING haplotypes and the risk of cervical precancerous lesions were estimated. No haplotypes in cGAS or STING gene was found to be significantly associated with risk of cervical precancerous lesions (Supplementary Table S5).

### Biological interaction of HPV infection and cGAS rs311678 for cervical precancerous lesions

We evaluated the interactive effects of HPV infection and each SNP based on an additive scale. According to the S index, a significant antagonistic interaction was found between cGAS rs311678 and HPV infection (S = 0.55, 95% CI = 0.32–0.96, *P* = 0.033) (Table 3).

**Table 3:** Results for gene-environment interaction analysis for each candidate SNP and HPV infection

Interaction group		Deviation from additive model	
	Gene	S (95% CI)	*P*^a^
1	rs610913*HPV infection	2.08 (0.41–10.59)	0.377
2	rs311678*HPV infection	0.55 (0.32–0.96)	**0.033**
3	rs4032697*HPV infection	0.57 (0.15–2.23)	0.414
4	rs311675*HPV infection	1.26 (0.46–3.45)	0.663
5	rs9352000*HPV infection	0.59 (0.22–1.59)	0.296
6	rs7761170*HPV infection	1.03 (0.56–1.90)	0.924
7	rs1131769*HPV infection	1.27 (0.62–2.63)	0.517
8	rs7380824*HPV infection	1.11 (0.63–1.95)	0.717
9	rs2516448*HPV infection	0.71 (0.43–1.17)	0.180

CI = confidence interval

a Adjusted for age(years) and the initial pregnancy of age(< 24 and ≥ 24 years). Bold values are statistically significant.

### MDR

According to the MDR selection model, a three-factor interaction model, containing HPV infection, age at menarche and rs311678 was optimal, with maximum CVC (10/10) and the highest TBA (71.64%) (significance test *P* = 0.001, and *P* for permutation test = 0.000–0.001) (Table 4).

**Table 4:** MDR models of GAS, STING and MHC gene and environmental factors of cervical precancerous lesions

Best Model	Training balanced accuracy	Testing balanced accuracy	Cross-validation Consistency	*P* value^a^
HPV infection	0.6957	0.6962	10/10	0.000–0.001
**HPV infection, age at menarche**	0.7088	0.6902	7/10	0.000–0.001
**HPV infection, age at menarche,rs311678**	**0.7451**	**0.7164**	**10/10**	**0.000–0.001**
HPV infection,age at menarche, genital cleansing after each intercourse,number of term birth	0.7989	0.5347	4/10	0.487

a 1000-fold permutation test. The best model(see text for details) are in bold.

### Three-way interaction between HPV infection, rs311678 status, and age at menarche with risk for cervical precancerous lesions

Based on the MDR model, we further performed a risk analysis of different combinations among the 3 factors (Table 5). The combination without any risk factors (including non-HPV-infected, wild-type for rs311678 and lower age at menarche: < 15 years) was used as a reference group. The individuals with a combination of 3 factors had a 4.27-fold greater risk of developing cervical precancerous lesions (OR = 4.27, 95% CI 1.81–10.04, *P* = 0.001), while the OR for cervical precancerous lesions in the presence of HPV infection, wild-type for rs311678 and increased age at menarche (≥ 15 years) was the highest (OR, 7.72; 95% CI, 3.39–17.57).

**Table 5:** Risk group analysis with 3 risk factors: HPV infection, age at menarche and rs311678

HPV infection	Age at menarche^b^	rs311678	Cases	Controls	OR (95% CI)	*P*^a^
−	−	−	10	82	1	
−	+	−	5	57	0.69 (0.22,2.12)	0.512
−	−	+	13	53	2.02 (0.82–4.95)	0.124
−	+	+	3	56	0.43 (0.11–1.65)	0.219
+	-	−	43	51	7.11 (3.27–15.44)	**< 0.001**
+	+	−	36	35	7.72 (3.39–17.57)	**< 0.001**
+	−	+	29	47	5.06 (2.26–11.33)	**< 0.001**
+	+	+	21	40	4.27 (1.81–10.04)	**0.001**

a Adjusted for age(years) and the initial pregnancy of age (< 24 and ≥ 24 years)

b Age at menarche(−, < 15years; +: ≥ 15 years). OR = odds ratio, CI = confidence interval. Bold values are statistically significant.

### Functional studies

**Figure 1:**
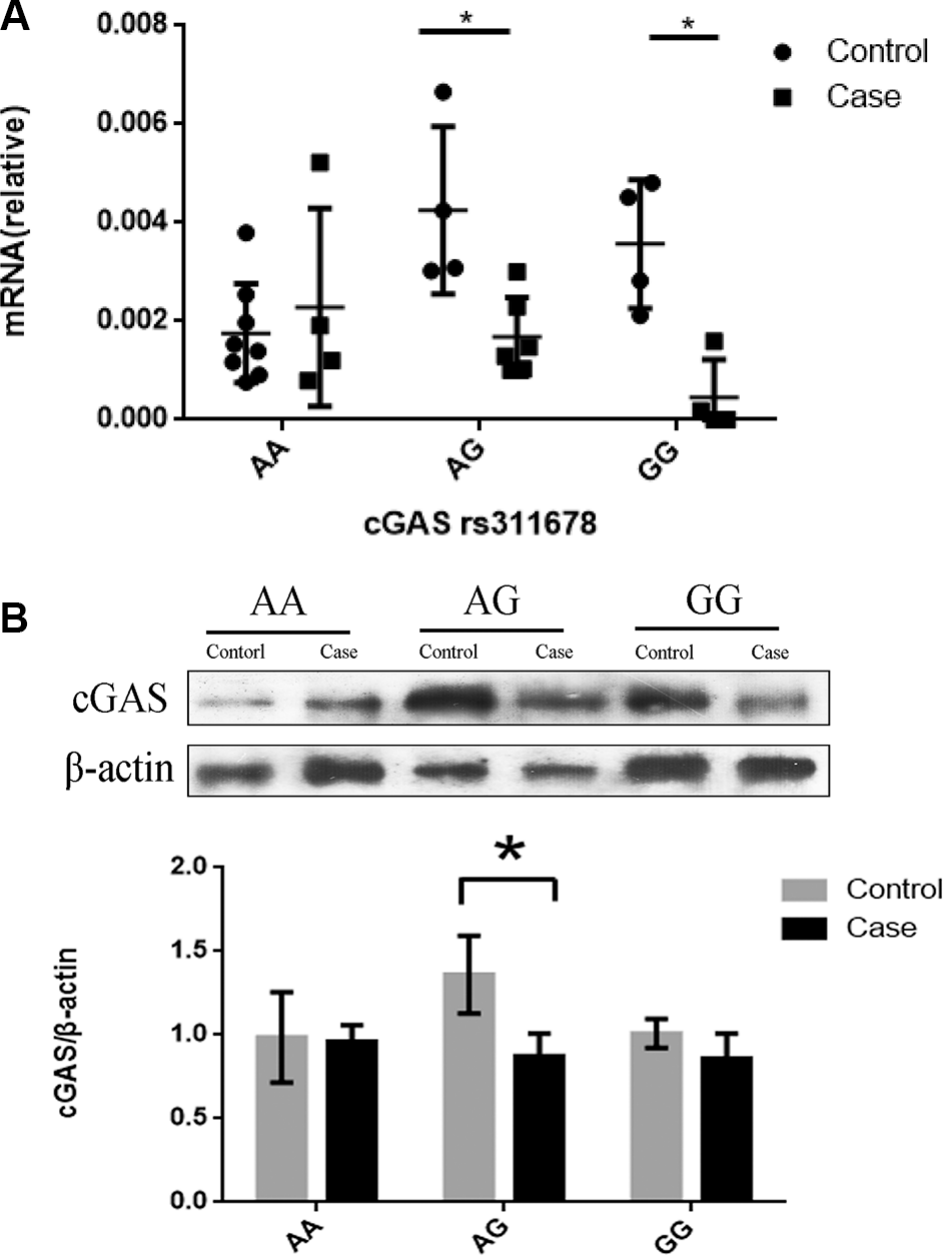
qRT-PCR, western blot analyses of cGAS rs311678 The results were expressed as the mean ± S.D. *P < 0.05. (**A**) The cGAS mRNA expression in SIL cases and controls by cGAS rs311678. (**B**) Western blotting analysis for cGAS rs311678 in human PBMCs.

To further study the functions of rs311678 in cGAS gene, thirty samples involved in 14 cases and 16 controls were randomly selected from our samples. As showed in Figure 1A, the AG and GG genotype in cGAS rs311678 had the lower mRNA expression levels in the SIL group, compared with control group (for AG genotype, *P* = 0.010; for GG genotype, *P* = 0.029). However, there was no difference in cGAS mRNA expression between SIL cases and controls among AA genotype in rs311678 (*P* = 0.925). Figure 1B revealed that the expression of cGAS protein in rs311678 AG was higher in control group than in SIL group (*P* = 0.008). However, there were no differences in cGAS protein expression in AA and GG genotypes between SIL cases and controls (for AA genotype, *P* = 0.667; for GG genotype, *P* = 0.095).

## DISCUSSION

To our knowledge, this is the first study that describes the interactive effects of SNPs in cGAS and along with STING and HPV infection on the risk of cervical precancerous lesions. The roles of the cGAS-STING pathway and the MHC genes in the pathogenesis of cervical precancerous lesions are still unclear. To investigate whether SNPs in these molecules affected the occurrence of cervical precancerous lesions, our study comprehensively evaluated the associations between variants of the cGAS-STING pathway, the MHC and cervical precancerous lesions.

cGAS detects cytosolic DNA in a sequence-independent manner, which elicits the cGAS-STING pathway to prime innate immune responses to various DNA viruses [19, 29]. Anghelina D et al. [30] have identified that cGAS deficiency resulted in a reduced early antiviral innate response to viral infection. Therefore, with the capacity for DNA sensing, cGAS plays an important role in IFN-I responses against DNA viruses [12, 14, 31]. Notably, cGAS can in principle detect any DNA that invades the cytoplasm, bind to DNA and is activated by DNA, irrespective of its sequence [29]. Hence, HPV infection was considered rather than high-risk HPV or low-risk HPV type. rs311678 is located in the intron 3–4 region of the cGAS gene. In our study, the minor allele in cGAS rs311678 reduced the risk of cervical precancerous lesions and had an antagonistic interaction with HPV infection. Furthermore, the results of qPCR and western blotting were basically consistent with our work, in which G allele in cGAS rs311678 might reduce the risk of cervical precancerous lesions in functional analysis. It has been demonstrated that introns contribute to the regulation of gene expression [32, 33] and transcript processing [34]. Matsushita et al. [35] reported that rs4376531 at intron 17 of the ARHGEF10 gene affected its transcriptional activity, which ultimately resulted in an increased incidence of atherothrombotic stroke. Similarly, we hypothesized that the rs311678 variant in the cGAS gene might lead to a higher level of cGAS transcripts and higher cGAS-STING pathway activity, which may interfere with the process whereby HPV encodes antagonists for the evasion of innate immune detection of HPV viral DNA [10, 36], thereby leading to HPV clearance. Secondly, the structure of mouse cGAS bound to an 18bp dsDNA through two binding sites, forming a 2:2 complex, therefore, we hypothesized that G of rs311678 might influence these two DNA binding sites that increase the affinity with HPV DNA, resulting in the activation of cGAS and thereby enhancing IFN-β induction and ultimately contributing to a decreased risk of cervical precancerous lesions [37, 38]. It is noteworthy that the cGAS/STING cascade has been viewed as an adjuvant target for vaccine applications [16].On the other hand, better understanding of the battle between the virus and the human host will most certainly improve the prevention and treatment of all viral diseases [31]. We could expect to target the activation of the cGAS-STING pathway for the effective prevention of cervical precancerous lesions.

**Figure 2:**
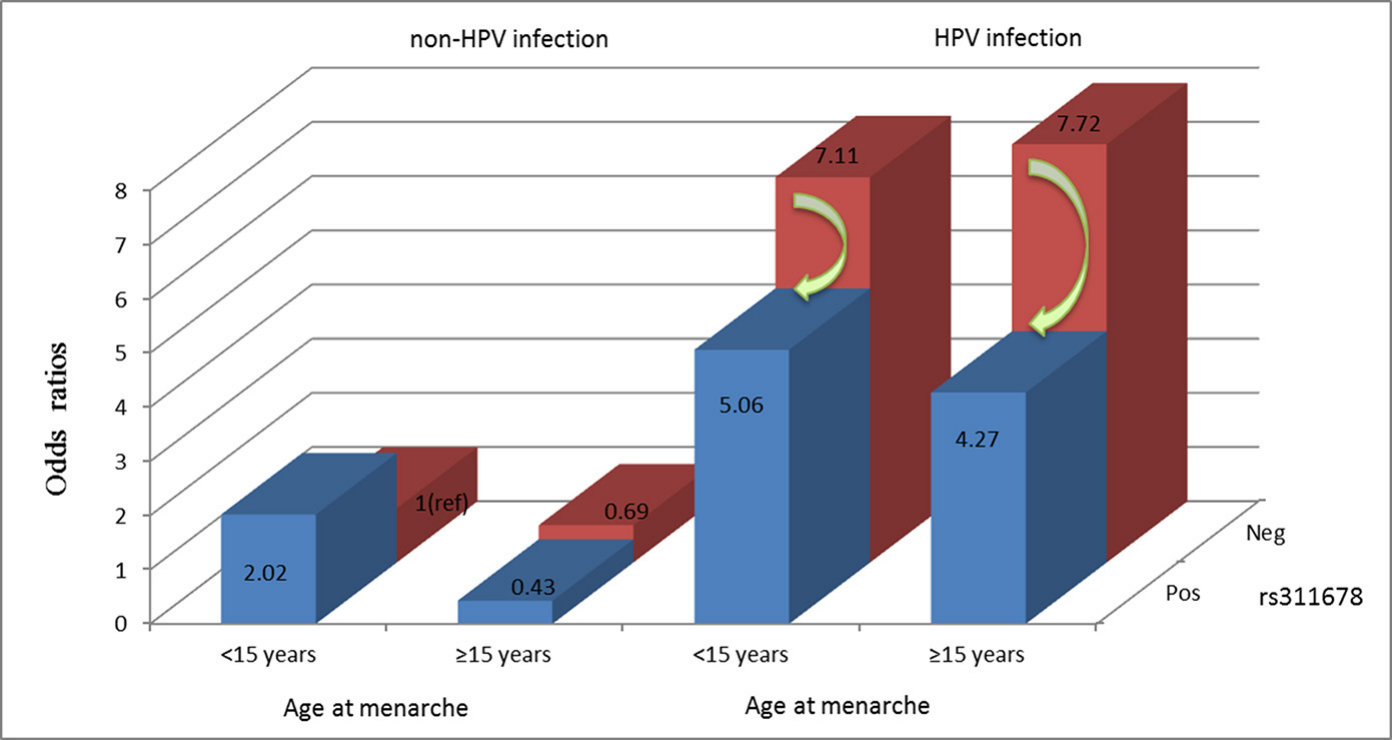
Risk analysis with 3 factors HPV infection, age at menarche and rs311678.

The present study statistically validated an interaction between HPV and each SNP in the cGAS-STING pathway in relation to the risk of cervical precancerous lesions, which is not enough. The MDR model showed that the three-locus model (i.e., HPV infection, age at menarche and rs311678) is the best model for the prediction of the risk of cervical precancerous lesions in our population. Notably, in our further study, the interaction among HPV infection, age at menarche and rs311678 was important at the population level; among HPV-infected individuals, women with age at menarche over 15 years and, carrying the minor G allele in cGAS rs311678 had a reduced risk of cervical precancerous lesions by 45% compared with individuals who were wild type at rs311678 (Figure 2).

rs2516448 is located 7.32 kb downstream of the MHC genes. Although it has been reported that the minor allele of rs2516448 increased the susceptibility to cervical cancer in Swedish women [27, 28], we did not find this association in Chinese women. One possible explanation could be that this association is relevant to only Caucasian populations and not to other ethnic groups. Further studies are needed to test the association between MHC rs2516448 and cervical precancerous lesions among those with non-Caucasian backgrounds [39]. In addition, variability in the frequency of HPV types, intratype variation, or host-virus interactions in the Chinese population may be responsible for the differences in association [38].

There are some potential limitations of this study. The main limitation of our study was the small number of SIL cases and controls, which might limit the likelihood of detecting weaker associations between SNPs and cervical precancerous lesions. A larger study would be required to address this issue. Also, we’d like to mention that the limited sample size of functional studies depended on availability of patients’ PBMCs. Another limitation is that the methods of calculating interactions based on the additive scale only apply to two factors at two levels, while the 95% confidence intervals of S were not calculated by the Excel spreadsheet when the variable factors were multiply variable [40].

In conclusion, our study provides evidence that the cGAS rs311678 polymorphism influences the risk of cervical precancerous lesions. Moreover, our study also finds that two-way and three-way gene-environment interactions exist in the etiology of cervical precancerous lesions. This study provides new epidemiological clues about the protective role of the cGAS-STING pathway in cervical precancerous lesions and further insights into such interactions in the etiology of cervical precancerous lesions. Furthermore, our results provide further evidence that introns should no longer be considered nonsense.

## MATERIALS AND METHODS

### Study sample

The study was approved by the Ethics Committee of School of Medicine in Jinan University. Cervical specimens were collected with a broom-like device (Qiagen, Valencia) and placed into a ThinPrep Pap test vial containing PreservCyt Solution. Referral Pap specimens were processed locally using the ThinPrep 2000 System (Hologic) and evaluated for routine screening cytology. A pap smear was positive for SIL if low (LSIL) and high (HSIL) grade squamous intraepithelial lesion, as classified according to the Bethesda Classification System, was detected [41]. Excluding other types of uterine diseases and no history of hysterectomy, a total of 164 cases were composed of 120 LSIL (73.2%) and 44 HSIL (26.8%). A total of 428 control samples without intraepithelial lesion or malignancy were recruited from the area of residence of the cases.

### HPV testing

Total DNA from cervical cells was extracted using a commercial magnetic beads kit (Chemagen; PekinElmer, Waltham, MA), according to the manufacturer's instructions. Then, 16 HPV types (6, 11, 16, 18, 31, 33, 35, 39, 45, 51, 52, 56, 58, 59, 66 and 68) were detected with the MassARRAY (Sequenom, San Diego, CA) technique based on matrix-assisted laser desorption/ionization time-of flight mass spectrometry [42]. All of the procedures were performed in the clinical standard laboratory of Beijing Genomics Institute.

### Genotype analysis

Tag single-nucleotide polymorphisms (SNPs) in the cGAS and STING genes were selected from the HapMap database (http://www.hapmap.org) according to the following selection strategy [43]. The screened region extended 10 kilobases upstream of the annotated transcription start site and downstream at the end of the last exon of each gene, which covered most of the genetic information in the Han Chinese in Beijing (CHB) population from the HapMap database (HapMap data rel 27 Phase + III, Feb09, on NCBI B36 assembly, dbSNP b126) [44]. tagSNPs were selected using a pairwise tagging algorithm setting the Hardy-Weinberg P-value, minor allele frequency (MAF) and r^2^ thresholds at 0.01, 0.01 and 0.8, respectively. The linkage disequilibrium (LD) pattern of each gene in the CHB population exhibited strong LD in several groups of tagSNPs (*r*^2^ ≥ 0.8), indicating that the most common SNPs can be captured by a subset of tagSNPs. Furthermore, we also selected SNPs based on previous findings from the literature. Eventually, 9 candidate SNPs were selected, including rs610913, rs311678, rs4032697, rs311675, rs9352000, rs7761170 in cGAS, rs1131769, rs7380824 in STING and rs2516448 in the MHC.

Genomic DNA from peripheral blood was extracted with phenol-chloroform DNA extraction methods. The samples were stored at −20°C until they were used. The DNA concentration was determined using a spectrophotometer (Nano Drop ND-1000, PerkinElmer, USA). Samples with a mean OD260 nm/OD280 nm of 1.8–2.0 and DNA concentration > 20 ng/μl were considered to be free of contamination.

Nine candidate SNPs were genotyped using a matrix-assisted laser desorption/ionization time of flight mass spectrometry (MALDI-TOF MS) method. Detail primer and amplified length for the determination of the 9 SNP genotypes is provided in Supplementary Table S2. The reaction mixture was desalted by adding resin, mixed, and resuspended in 16 μL of water. Once the primer extension reaction was completed, the samples were spotted onto target samples using a RS1000 MassARRAY Nanodispenser and genotyped using MALDI-TOF. Genotyping was performed in real time with Typer software version 4.0. Genotype sequencing was performed by Invitrogen Trading (Shanghai) Co., Ltd. The distribution of SNPs in the cGAS, STING and MHC genes is shown in Supplementary Table S3.

### Quantitative real-time reverse transcription PCR (qRT-PCR) analysis

The peripheral blood mononuclear cells (PBMCs) were isolated from EDTA-anticoagulated blood with Ficoll–Hypaque density-gradient centrifugation. Total RNA was extracted from PBMCs using TRIzol (Invitrogen, Carlsbad, CA), followed by reverse transcription using a transcriptase cDNA kit (Takara-PrimeScript RT Master Mix kit, Otsu, Japan). Then we finished qRT-RCR analysis to quantify the mRNA expression of cGAS with the SYBR PrimerScript RT-PCR kit (TaKaRa, Otsu, Japan) normalized to mRNA β-actin. The assays were performed on a Bio-Rad's CFX96 real-time system (Bio-Rad Laboratories). Cycle conditions were 95°C for 30s followed by 45 cycles at 95°C for 5s and 60°C for 30s. Relative expression levels were calculated using the 2^−^^ΔΔCt^ method. The data of the two independent analyses for each sample and parameters were averaged and relative expression levels were presented as the relative fold change analyzed using unpaired two-tailed *t* test.

### Western blot

PBMCs were homogenized in RIPA Lysis Buffer at 4°C, and then centrifuged 10 min in 13,000 × g at 4°C. Protein concentrations of the homogenates were determined by BCA kit. The proteins were separated by 8% sodium dodecy1 sulface polyacrylamide gel electrophoresis (SDS-PAGE) and electrotransferred to Immobilon-P membrane (Millipore, Bedford, MA, USA). The primary antibody (Supplementary Table S1) at 4°C overnight and then with a corresponding anti-rabbit IgG conjugated to horseradish peroxidase (1 : 5000) at 37°C for 1 h. Immunoreactive bands were visualized with a chemiluminescent substrate (ECL) kit.

### Statistical analysis

Logistic regression was used to calculate odds ratios (ORs) and their relative 95% confidence intervals (CIs) for risk estimation. A chi-square test was used to evaluate the Hardy-Weinberg equilibrium and the dependence of the allele frequencies between the cases and controls. In addition, a *t*-test or chi-square test was used to examine differences in the demographic characteristics and HPV infection status between the cases and controls.

Based on the observed genotypes, haplotype frequencies and effects were examined using the SHEsis, a powerful software platform (http://analysis.bio-x.cn/myAnalysis.php) for analyses of haplotype construction [45]. The global score test was used to estimate the overall differences in haplotype frequencies between cases and controls. The estimated ORs and 95% CI were also used to estimate the effects of individual haplotypes on cervical precancerous lesions.

We explored the additive interaction between the factors according to the following strategy [46]. Among cases and controls, a binary classification was used both for HPV infection (infection vs. non-infection) and for genotypes (homozygous for the major allele vs. one or two copies of the minor allele). The risk for cervical precancerous lesions for a given SNP and HPV infection status was expressed by OR_i,,j_ where the first index (i) indicated the HPV infection status coded as 0 for non-infected and 1 for infected subjects, and the second index (j) indicated the SNP genotype, coded as 0 for subjects homozygous for the major allele and 1 for subjects bearing one or two copies of the minor allele. Subjects who were HPV-uninfected and homozygous for the major allele were considered the reference group, thus coding their cervical precancerous lesions risk as OR_00_ = 1. The relative ORs were obtained by logistic regression. The confidence intervals were calculated by the regression coefficients and the corresponding covariance matrix [47]. Deviation from an additive model was calculated as the relative excess risk due to S (the synergy index). Biological interactions in the regression models were tested as departure from additivity. Based on the adjusted odds ratios obtained in the logistic regression models, an Excel spreadsheet (www.epinet.se) was used to calculate S on an additive scale and its corresponding confidence intervals [47]. An S value (95% CI) that does not cross 1 indicates a biological interaction [48, 49]. In addition, S > 1 indicates synergetic effects and S < 1 indicates antagonistic effects [50, 51].

Finally, the multifactor dimensionally reduction (MDR) method [52–55] was applied to analyze high-order gene-gene and gene-environment interactions. To search for the best n-factor model, the data were divided into 10 sets: 1 for testing and 9 for training. In brief, the multilocus genotypes were pooled into high-risk and low-risk groups, effectively reducing the genotype predictors to one dimension. The result was a set of models, and the Testing Balanced Accuracy (TBA) and cross-validation consistency (CVC) indexes were used to determine the overall best model. An MDR-pt procedure was used to evaluate the significance of the selected models via calculating empirical 1000-fold permutation tests .The model with the maximum TBA and CVC and a *P* value for TBA in MDR-pt results less than 0.05 was considered the best model.

MDR software v.3.0.2 and MDR permutation testing software (version 1.0 beta 2) were used in this study and were freely available online (www.epistasis.org). All other statistical analyses were performed using SPSS software v.16.0 (SPSS, Inc.). Significant associations were defined as *P* < 0.05.

## SUPPLEMENTARY MATERIALS FIGURES AND TABLES


