# Supplementary material for: Interaction between susceptibility loci in cGAS-STING pathway, MHC gene and HPV infection on the risk of cervical precancerous lesions in Chinese population
Source: Oncotarget. 2016 Oct 1;7(51):84228–38. doi: 10.18632/oncotarget.12399 (PMC5356657; doi:10.18632/oncotarget.12399)
Supplement: Supplementary file 1 [file oncotarget-07-84228-s001.pdf]

Interaction between susceptibility loci in cGAS-STING pathway, MHC gene and HPV infection on the risk of cervical precancerous lesions in Chinese population

Supplementary Materials

Supplementary Table S1: Information of the primary antibodies used in this study

Anitibody	Specificity	Type	Dilution[a]	Source
C6orf150	hcGAS	Polyclonal	1:1000 WB	Abcam
β-actin	β-actin	monoclonal	1:1000 WB	Cell Signaling

[a]WB, western blot.

Supplementary Table S2: Primer sequences and amplified length of 9 SNPs in cGAS, STING and MHC gene

Gene	SNP	Primers	Amplified length (bp)
cGAS	rs610913	F:ACGTTGGATGGACAGCATCTTAGAAGCTGA	126
		R:ACGTTGGATGCCAACACTCGTGCATATTAC	
	rs311678	F:ACGTTGGATGCTGGAAACTCATTGTTTCT	100
		R:ACGTTGGATGCTCTAGCAACTTAATTGAC	
	rs4032697	F:ACGTTGGATGGTCATGTCTCCTTAAGCTAC	96
		R:ACGTTGGATGATACCTAACTAGTACTCCTC	
	rs311675	F:ACGTTGGATGCCCCGTACTTCCAAGACTTT	120
		R:ACGTTGGATGAGATGGTGGTCTCCCTTTGT	
	rs9352000	F:ACGTTGGATGCTTCCGCACGGAATGCCAG	144
		R:ACGTTGGATGTTTTTCTGCCGGGATCCCGA	
	rs7761170	F:ACGTTGGATGGATTTCTGGATGTCAGTAGC	86
		R:ACGTTGGATGAGGAGACTATGAGTGTTTTG	
STING	rs1131769	F:ACGTTGGATGTATGGCTGACCCCAACATTC	106
		R:ACGTTGGATGTGCTGTAAACCCGATCCTTG	
	rs7380824	F:ACGTTGGATGAGTTGTTCTGAGACTCAGGG	93
		R:ACGTTGGATGCTTGAGCAGGCCAAACTCT	
MHC	rs2516448	F:ACGTTGGATGTCTCCTCCCTTTTTGCATCC	130
		R:ACGTTGGATGCTTCATACATACACACACAC	

Supplementary Table S3: Distribution of SNPs in cGAS, STING and MHC genes

SNP ID	Position	Functional region	Major/minor allele	MAF%	P(HWE)
cGAS(6q13)					
rs610913	74212067	missense	C/A	52.4	0.886
rs311678	74191737	intron	A/G	27.7	0.268
rs4032697	74200984	intron	A/G	3.5	0.463
rs311675	74194190	intron	T/A	5.1	0.271
rs9352000	74218522	missense	T/G	5.3	0.250
rs7761170	74209619	intron	G/T	19.2	0.946
STING(5q31.2)					
rs1131769	138838103	missense	G/A	9.5	0.902
rs7380824	138837166	missense	C/T	42.8	0.544
MHC(6q21.3)					
rs2516448	31498389	Nearby MICA gene	G/A	20.0	0.251

MAF=Minor allele frequency, HWE=Hardy-Weinberg equilibrium.

Supplementary Table S4: The association between genotypes of cGAS and STING and cervical precancerous lesions

Gene name	SNP	Genotype	Cases *N* (%)	Controls *N* (%)	Crude OR (95% CI)	*P*	Adjusted OR (95% CI)	*P*
cGAS	rs610913	CC	41(25.3%)	95(22.5%)	1(Ref)		1(Ref)	
		CA	76(46.9%)	212(50.2%)	0.83(0.53-1.30)	0.419	0.84(0.53-1.32)	0.439
		AA	45(27.8%)	115(27.3%)	0.91(0.55-1.50)	0.703	0.91(0.55-1.51)	0.713
	Dominant	CA+AA	121(74.7%)	327(77.5%)	0.86 (0.56-1.31)	0.474	0.86 (0.56-1.32)	0.493
	Recessive	CC+CA	117(72.2%)	307(72.7%)	1(Ref)		1(Ref)	
		AA	45(27.8%)	115(27.3%)	1.03(0.69-1.54)	0.898	1.03(0.68-1.54)	0.903
	rs311678	AA	95(59.0%)	225(53.3%)	1(Ref)		1(Ref)	
		AG	60(37.3%)	160(37.9%)	0.89(0.61-1.30)	0.542	0.90(0.62-1.33)	0.600
		GG	6(3.7%)	37(8.8%)	0.38(0.16-0.94)	**0.036**	0.40(0.16-0.98)	**0.045**
	Dominant	AG+GG	66(41.0%)	197(46.7%)	0.79(0.55-1.15)	0.218	0.81(0.56-1.17)	0.262
	Recessive	AA+AG	155(96.3%)	385(91.2%)	1(Ref)		1(Ref)	
		GG	6(3.7%)	37(8.8%)	0.40(0.17-0.97)	**0.043**	0.41(0.17-1.01)	0.052
	rs4032697	AA	153(95.0%)	389(93.1%)	1(Ref)		1(Ref)	
		AG	8(5.0%)	29(6.9%)	0.70(0.31-1.57)	0.388	0.72(0.32-1.62)	0.424
	Dominant	AG+GG	8(5.0%)	29(6.9%)	0.71(0.31-1.57)	0.388	0.72(0.32-1.62)	0.424
	rs311675	TT	150(91.5%)	380(89.8%)	1(Ref)		1(Ref)	
		TA	14(8.5%)	43(10.2%)	0.83(0.44-1.55)	0.550	0.83(0.44-1.58)	0.577
	Dominant	TA+AA	14(8.5%)	43(10.2%)	0.83(0.44-1.55)	0.550	0.83(0.44-1.58)	0.577
	rs9352000	TT	147(90.2%)	381(89.4%)	1(Ref)		1(Ref)	
		TG	16(9.8%)	45(10.6%)	0.92(0.51-1.68)	0.790	0.90(0.49-1.65)	0.740
	Dominant	TG+GG	16(9.8%)	45(10.6%)	0.92(0.51-1.68)	0.790	0.90(0.49-1.65)	0.740
	rs7761170	GG	109(66.5%)	278(65.3%)	1(Ref)		1(Ref)	
		GT	45(27.4%)	132(31.0%)	0.87(0.58-1.30)	0.498	0.87(0.58-1.31)	0.513
		TT	10(6.1%)	16(3.8%)	1.59(0.70-3.62)	0.265	1.63(0.71-3.76)	0.252
	Dominant	GT+TT	55(33.5%)	148(34.7%)	0.95(0.65-1.39)	0.783	0.95(0.65-1.40)	0.806
	Recessive	GG+GT	154(93.9%)	410(96.2%)	1(Ref)		1(Ref)	
		TT	10(6.1%)	16(3.8%)	1.66(0.74-3.75)	0.219	1.70(0.74-3.88)	0.209
STING	rs1131769	GG	132(80.5%)	347(82.0%)	1(Ref)		1(Ref)	
		GA	30(18.3%)	72(17.0%)	1.10(0.68-1.75)	0.705	1.12(0.70-1.80)	0.641
		AA	2(1.2%)	4(0.9%)	1.31(0.24-7.26)	0.754	1.30(0.23-7.28)	0.767
	Dominant	GA+AA	32(19.5%)	76(18.0%)	1.11(0.70-1.75)	0.665	1.13(0.71-1.80)	0.607
	Recessive	GG+GA	162(98.8%)	419(99.1%)	1(Ref)		1(Ref)	
		AA	2(1.2%)	4(0.9%)	1.29(0.24-7.13)	0.768	1.28(0.23-7.12)	0.784
	rs7380824	CC	49(30.1%)	142(33.4%)	1(Ref)		1(Ref)	
		CT	86(52.8%)	202(47.5%)	1.23(0.82-1.86)	0.317	1.26(0.83-1.90)	0.282
		TT	28(17.2%)	81(19.1%)	1.00(0.59-1.72)	0.995	1.02(0.60-1.76)	0.935
	Dominant	CT+TT	114(69.9%)	283(66.6%)	1.17(0.79-1.73)	0.438	1.19(0.80-1.76)	0.389
	Recessive	CC+CT	135(82.8%)	344(80.9%)	1(Ref)		1(Ref)	
		TT	28(17.2%)	81(19.1%)	0.88(0.55-1.42)	0.599	0.89(0.55-1.43)	0.630
MHC	rs2516448	GG	104(63.4%)	266(63.2%)	1(Ref)		1(Ref)	
		GA	53(32.3%)	142(33.7%)	0.96(0.65-1.41)	0.815	0.97(0.65-1.43)	0.864
		AA	7(4.3%)	13(3.1%)	1.38(0.54-3.55)	0.507	1.35(0.52-3.48)	0.539
	Dominant	GA+AA	60(36.6%)	155(36.8%)	0.99(0.68-1.44)	0.958	1.00(0.69-1.46)	0.997
	Recessive	GG+GA	157(95.7%)	408(96.9%)	1(Ref)		1(Ref)	
		AA	7(4.3%)	13(3.1%)	1.40(0.55-3.57)	0.482	1.36(0.53-3.49)	0.519

[a]Adjusted for age (years) and the initial pregnancy of age (< 24 and ≥ 24 years). OR = odds ratio, CI = confidence interval. Bold values are statistically significant.

Supplementary Table S5: The haplotype frequencies of cGAS and STING polymorphisms and cervical precancerous lesions

Gene	Haplotype	Haplotype frequency		OR (95% CI)	*P*
		cases	controls		
cGAS					
	A A A T T G[a]	141 (0.445)	354 (0.428)	1.00	
	A G A A T G[a]	14 (0.044)	42 (0.051)	0.84 (0.44–1.58)	0.583
	A G A T T G[a]	9 (0.029)	33 (0.040)	0.69 (0.32–1.47)	0.331
	C A A T G G[a]	16 (0.051)	42 (0.051)	0.96 (0.52–1.76)	0.885
	C A A T T G[a]	43 (0.135)	103 (0.125)	1.05 (0.70–1.57)	0.821
	C A A T T T[a]	47 (0.150)	104 (0.126)	1.14 (0.76–1.69)	0.531
	C G A T T G[a]	24 (0.077)	63 (0.076)	0.96 (0.58–1.59)	0.862
	C G A T T T[a]	15 (0.046)	57 (0.069)	0.66 (0.36–1.21)	0.177
	C G G T T G[a]	6 (0.019)	26 (0.031)	0.58 (0.23–1.44)	0.239
Global test	0.710				
STING					
	G C[b]	150 (0.460)	406 (0.481)	1.00	
	GT[b]	142 (0.436)	359 (0.426)	1.07 (0.82–1.40)	0.619
	A C[b]	34 (0.104)	77 (0.091)	1.20 (0.77–1.87)	0.433
Global test	0.713				

[a]cGAS haplotypes: rs610913-rs311678-rs4032697-rs311675-rs9352000-rs7761170; [b]STING haplotype: rs1131769-rs7380824. OR = odds ratio, CI = confidence interval.
